# Metabolic Complications During the Initial Management of Diabetic Ketoacidosis According to Diabetes Type in a Single Tertiary Centre in Malaysia

**DOI:** 10.7759/cureus.101129

**Published:** 2026-01-08

**Authors:** Zi Yang Lian, Jeffrey Soon-Yit Lee, Ijaz Hallaj Rahmatullah, Shanty Velaiutham

**Affiliations:** 1 Endocrine Unit, Department of Medicine, Hospital Pulau Pinang, Ministry of Health Malaysia, Penang, MYS; 2 Clinical Research Centre, Hospital Sibu, Ministry of Health Malaysia, Sarawak, MYS; 3 Endocrine Unit, Department of Medicine, Hospital Raja Permaisuri Bainun, Ministry of Health Malaysia, Perak, MYS

**Keywords:** diabetes ketoacidosis, hypoglycemia, hypokalemia, risk predictors, type 1 diabetes mellitus, type 2 diabetes mellitus

## Abstract

Introduction: Diabetic ketoacidosis (DKA) is traditionally associated with type 1 diabetes mellitus (T1DM) but is increasingly observed in type 2 diabetes mellitus (T2DM). Despite notable pathophysiological differences between these conditions, current DKA management follows standardized protocols regardless of diabetes classification. This “one-size-fits-all” approach raises concerns about potential treatment-related metabolic complications that are specific to distinct diabetes phenotypes.

Objectives: To compare the rates of metabolic complications - specifically hypoglycemia and hypokalemia - during the initial management of DKA between T1DM and T2DM patients and identify associated factors. Secondary outcomes included evaluating the length of hospital stay (LOS), complication rates, and in-hospital mortality, and identifying predictors of hypoglycemia and hypokalemia during DKA treatment.

Methods: A retrospective cohort study of DKA admissions at Penang General Hospital, Malaysia, was conducted between 2019 and 2023. Data on DKA admissions, metabolic complications, and hospitalization outcomes were extracted and analysed.

Results: We reviewed the case records of 215 DKA admissions: 100 (46.5%) T1DM, 115 (53.5%) T2DM. The prevalence of hypoglycemia was significantly higher in patients with T1DM compared to T2DM (33/100, 33.0% vs 18/115, 15.7%, *p*=0.003). Conversely, the prevalence of hypokalemia did not differ significantly between the groups (55/100, 55.0% vs 62/115, 53.9%, *p*=0.873). Multivariate analysis identified T1DM and low body weight as independent predictors of hypoglycemia. Independent predictors for hypokalemia included low pH, low initial serum potassium, shorter diabetes duration, and absence of nephropathy. Patients with T1DM had a lower observed mortality rate (0/100, 0.0% vs 4/115, 3.5%) and fewer 30-day readmissions (4/100, 4.0% vs 10/115, 8.7%) compared to T1DM, but not statistically significant. T1DM patients required more intensive care, whereas T2DM patients experienced significantly longer hospital stays and higher rates of nosocomial infection.

Conclusion: Significant differences exist in both metabolic safety profiles and clinical trajectories when managing T1DM and T2DM patients with DKA. While T1DM patients are prone to hypoglycemia, T2DM patients carry a higher burden of comorbidities leading to prolonged hospitalization and infection risks. Tailoring DKA management strategies specifically to diabetes type may reduce treatment-related complications and improve overall clinical outcomes.

## Introduction

Diabetic ketoacidosis (DKA) is a medical emergency characterized by hyperglycemia, dehydration, metabolic acidosis, and increased body ketone production, which is associated with significant morbidity and mortality [1,2]. Historically, DKA was considered a hallmark feature distinguishing individuals with type 1 diabetes mellitus (T1DM) from those with type 2 diabetes mellitus (T2DM) [3,4]. This distinction was based on pathophysiological differences - marked insulin deficiency in T1DM, versus predominant insulin resistance in T2DM [3,4].

However, DKA has been increasingly observed in T2DM in recent years, paralleling the rising prevalence of T2DM globally [4-6]. Notably, one study reported that up to 61.9% of patients admitted for DKA had T2DM [7]. The management of DKA in individuals with T2DM remains identical to that of those with T1DM, despite differences in their clinical phenotype, which include greater body weight, insulin resistance, older age, and increased comorbidities in the T2DM population [3,4]. Some studies reported patients with T2DM may exhibit varied clinical and biochemical presentations, such as a tendency for initial normal serum potassium and less pronounced acidosis, likely due to residual insulin reserve [4,5]. Despite these variations, current guidelines do not differentiate between these two groups.

The Joint British Diabetes Societies for inpatient care (JBDS-IP) guidelines and the consensus report on hyperglycemic crises advocate the use of fixed-rate intravenous insulin infusion (FRIII) started at 0.1 units/kg/hour, regardless of diabetes type [1,8]. It remains unclear whether applying this undifferentiated management approach to physiologically distinct groups impacts clinical outcomes and safety [3].

Hypoglycemia and hypokalemia are common iatrogenic metabolic complications arising from intravenous insulin infusion during DKA treatment [1-3]. Previous studies indicate that these complications are more prevalent in patients receiving high doses of insulin [1,2]. Hypoglycemia in hospitalized patients is associated with increased length of hospital stay, morbidity, and mortality [2,9]. Hypokalemia can lead to respiratory muscle weakness or fatal arrythmias, and intravenous insulin therapy may need to be delayed until serum potassium is corrected [2]. Thus, identification of risks for hypoglycemia and hypokalemia during DKA treatment is crucial to improve patient outcomes.

The rates of metabolic complications between T1DM and T2DM under standardized protocols remain insufficiently studied [3]. There are a few small studies reporting these complications, but data are conflicting. Ooi et al. discovered hypoglycemia occurred more frequently in T1DM with no differences in hypokalemia, whereas Eledrisi et al. demonstrated similar hypoglycemia rates but higher hypokalemia in T2DM [3,6,10].

The primary objective of this study is to compare the metabolic complications rates - specifically hypoglycemia and hypokalemia - between patients with T1DM and T2DM managed under standard DKA protocols. Secondary objectives are to evaluate the impact of diabetes type on hospital length of stay, complication rates, and in-hospital mortality, and to identify predictors of hypoglycemia and hypokalemia during DKA treatment.

## Materials and methods

**Figure 1:**
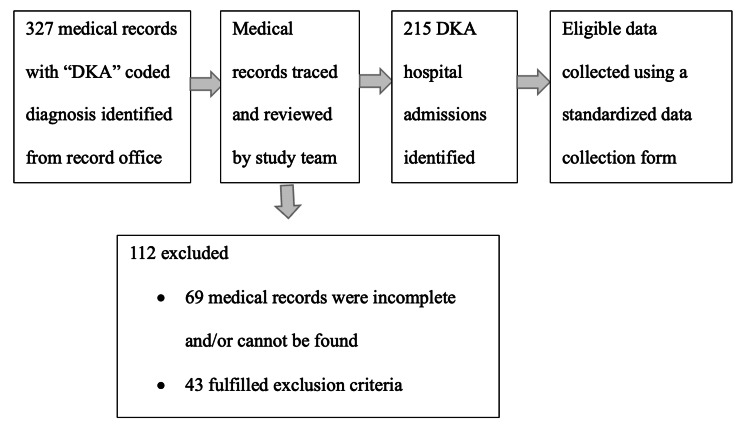
Cohort flow chart illustrating patient selection process. DKA: diabetic ketoacidosis

Data were collected retrospectively from paper and electronic medical records of all DKA admissions at Penang General Hospital, a tertiary specialist referral centre serving approximately 1.7 million people in northern peninsular Malaysia (Figure 1). 

In this hospital, all DKA patients were managed following the JBDS-IP guidelines [1]. The study period spanned from 1st January 2019 to 31st December 2023. We reviewed the medical records of patients with a confirmed diagnosis of DKA as defined by the JBDS-IP guidelines who had either T1DM or T2DM [1,11]. T1DM was defined by the presence of diabetes mellitus autoantibodies (e.g., anti-GAD, anti-IA2, anti-ICA), low C-peptide levels, exclusive insulin treatment since diagnosis, or clinician-diagnosed T1DM. T2DM patients were defined by phenotypic indicators of insulin resistance (e.g. overweight, obesity, acanthosis nigricans), a family history of diabetes mellitus, treatment with oral glucose-lowering drugs (OGLDs) or diet alone, and/or negative autoimmune antibody result. Repeat admissions for DKA were included and analysed as independent clinical events. Exclusion criteria included euglycemic DKA induced by oral glucose-lowering drugs (specifically sodium-glucose co-transporter 2 (SGLT2) inhibitors), pregnancy, end-stage renal failure on dialysis, unclassified newly diagnosed diabetes mellitus, confirmed genetic hypokalemia syndromes (e.g. Bartter’s syndrome, Gitelman’s syndrome, familial hypokalemia periodic paralysis, primary aldosteronism, congenital adrenal hyperplasia, etc.), and admissions under paediatric care. Only case records with complete data were included in the review (Figure 1).

Baseline data (e.g. age, gender, ethnicity, weight, height, body mass index (BMI)), clinical and laboratory data (e.g. diabetes type, duration of diabetes diagnosis during admission, presence of comorbidities, medication, diabetes complications, vital signs, blood sugar, blood gases, serum or urine ketone, presenting symptoms), and primary outcomes (occurrence of hypoglycemia is defined by capillary blood glucose level less than 4.0mmol/L, and hypokalemia is defined by serum potassium of less than 3.4mmol/L) until DKA resolution (defined as serum ketone <0.6mmol/L and venous pH >7.3) or during the first 48 hours of DKA treatment, whichever occurred earlier were recorded. Secondary outcomes included in-hospital average length of stay (LOS), complications, and mortality. Data extraction was performed by a single investigator using a standardized data collection form to minimize inter-observer variability. 

Statistical analysis

Sample size was calculated to detect a proportional difference in prevalence between T1DM and T2DM, with 80% power. The prevalence of hypoglycemia and hypokalemia in T1DM was 76.9% and 58.0%, respectively, based on a study by Balmier et al. [3]. To detect the smallest important differences of 27.0% in hypoglycemia and 24.0% in hypokalemia, we required 64 T1DM and 64 T2DM participants. To compensate for potential missing information or participants, the sample size was inflated by 10% to 72 T1DM and 72 T2DM participants. Numerical data were presented as mean and standard deviation (SD) for normally distributed data, and median and interquartile range (IQR) for skewed data. Numerical data comparison was done using the independent t-test or Mann-Whitney U test as appropriate. Categorical data were presented using frequency and percentages, and inferential analysis was performed using Chi-Square or Fisher’s Exact test. Predictors of complications were identified using univariate and multivariate logistic regression (forward likelihood ratio (LR) method), with model performance evaluated via the Hosmer-Lemeshow test and Pseudo R2. Statistical significance was set at p value less than 0.05. Statistical analysis was performed using SPSS Statistics version 29 (IBM Corp., Armonk, NY, USA).

## Results

A total of 155 patients were included, contributing to 215 unique DKA admissions: 100 (46.5%) T1DM and 115 (53.5%) T2DM. The cohort was predominantly Malay and female. Participants with T1DM were younger, weighed less, and had a lower BMI compared to those with T2DM (Table 1). The majority of T1DM patients were on insulin alone, while most T2DM patients were on OGLDs.

**Table 1:** Baseline characteristics of subjects. A total of 155 patients were included, contributing to 215 unique diabetic ketoacidosis (DKA) admissions. IQR: interquartile range; BMI: body mass index; DM: diabetes mellitus; OGLD: oral glucose lowering drug. *: Includes patients from Myanmar, Bangladesh, Thailand, and Indonesia. ^*^Mann-Whitney test; ^†^ Pearson Chi-Square; ^‡^ Fisher’s Exact test

Baseline characteristics		Type 1 Diabetes N = 62	Type 2 Diabetes N = 93	p value
Median (IQR) age (years)		28.0 (6.0)	56 (29.9)	<0.001^*^
Gender (n,%)	Female	40 (64.5)	50 (53.8)	0.184^†^
	Male	22 (35.3)	43 (46.2)	
Ethnicity (n,%)	Malay	30 (48.4)	50 (53.8)	
	Chinese	19 (30.6)	23 (24.7)	
	Indian	10 (16.1)	18 (19.4)	
	Other*	3 (4.8)	2 (2.2)	
Median (IQR) weight (kg)		51.8 (15.6)	60.0 (5.0)	0.008^*^
Median BMI (kg/m^2^)		20.7 (6.6)	24.5 (7.2)	0.001^*^
DM duration (n,%)	<10 years	35 (56.5)	50 (53.8)	0.742^†^
	>10 years	27 (43.5)	43 (46.2)	
Comorbid (n,%)	None	46 (74.2)	36 (38.7)	
	Hypertension	4 (6.5)	17 (18.3)	
	Dyslipidaemia	7 (11.3)	12 (12.9)	
	Hypertension and dyslipidaemia	5 (8.1)	28 (30.1)	
Medications (n,%)	Insulin and OGLD	54 (87.1)	73 (78.5)	
	Insulin	54 (87.1)	52 (55.9)	
	OGLD	17 (27.4)	65 (69.9)	
	Poor-adherence to medications	1 (1.6)	9 (9.7)	
	Newly diagnosed DM	7 (11.3)	11 (11.8)	
	Diuretics	0 (0.0)	5 (5.4%)	
Microvascular complications (n,%)	Retinopathy	5 (8.1)	4 (4.3)	0.485^‡^
	Neuropathy	2 (3.2)	1 (1.1)	0.564^‡^
	Nephropathy	13 (21.0)	38 (40.9)	0.010^†^
Macrovascular complications (n,%)	Cardiovascular disease	2 (3.2)	15 (16.1)	0.012^†^
	Cerebrovascular disease	1 (1.6)	15 (16.1)	0.004^†^
	Peripheral arterial disease	2 (3.2)	11 (11.8)	0.058^†^

Poor adherence to medications was the predominant precipitant for DKA admissions in T1DM, whereas T2DM DKA were more commonly precipitated by infection, poor adherence, or a combination of both. Symptoms differed: T1DM patients presented more frequently with gastrointestinal symptoms (vomiting, abdominal pain), while cough, higher blood pressure, and oxygen requirement were more common in T2DM. There were no significant differences in admission heart rate, temperature, total insulin dose, or time to DKA resolution between the groups (Table 2).

**Table 2:** Clinical characteristics on admission. Clinical characteristics of 215 admissions (a total of 155 patients were included, contributing to 215 unique DKA admissions). SD: standard deviation; IQR: interquartile range; DM: diabetes mellitus; DKA: diabetic ketoacidosis; SBP: systolic blood pressure; DBP: diastolic blood pressure; HR: heart rate; CBS: capillary blood glucose; IV: intravenous *: Includes stroke, transient ischemic attack, recent surgery, gastrointestinal bleeding ^†^Independent t-test; ^‡^Mann-Whitney test; ^§^Pearson Chi-Square; ^‖^Fisher’s Exact test

Clinical characteristics on admission		Type 1 Diabetes N=100	Type 2 Diabetes N=115	p value
Mean (SD) SBP (mmHg)		125 (19.7)	134 (22.9)	0.004^†^
Mean (SD) DBP (mmHg)		78 (16.8)	80 (17.6)	0.266^†^
Mean (SD) HR (beats per minute)		116 (19.4)	116 (20.5)	0.930^†^
Median (IQR) body Temperature (^o^C)		36.9 (0.50)	36.9 (1.10)	0.519^‡^
Mean (SD) CBG(mmol/L)		30 (10.0)	32 (13.2)	0.129^†^
Mean (SD) serum Potassium (mmol/L)		5.0 (0.95)	5.1 (0.99)	0.405^†^
Mean (SD) serum Creatinine (µmol/L)		103 (52.0)	123 (86.0)	<0.001^†^
Mean (SD) admission pH		7.07 (0.140)	7.10 (0.141)	0.068^†^
Mean (SD) admission bicarbonate		9.30 (3.421)	10.00 (3.082)	0.115^†^
Mean (SD) serum ketone		6.4 (1.57)	6.6 (1.60)	0.410^†^
Mean (SD) admission HbA1c (%)		12.4 (2.75)	12.4 (3.17)	0.973^†^
Median (IQR) initial IV insulin (units/hour)		5.5 (2.00)	6.0 (1.00)	<0.001^‡^
Median (IQR) total insulin (units) until DKA resolution		96 (58.8)	90 (78.0)	0.655^‡^
Symptoms (n,%)				
Polyuria		14 (14.0)	16 (13.9)	0.985^§^
Polydipsia		15 (15.0)	13 (11.3)	0.422^§^
Polyphagia		4 (4.0)	1 (0.9)	0.186^‖^
Weight loss		6 (6.0)	6 (5.2)	0.803^§^
Nausea		18 (18.0)	13 (11.3)	0.163^§^
Vomiting		76 (76.0)	64 (55.7)	0.002^§^
Abdominal pain		41 (41.0)	26 (22.6)	0.004^§^
Fever		18 (18.0)	25 (21.7)	0.494^§^
Lethargy		43 (43.0)	62 (53.9)	0.110^§^
Breathlessness		36 (36.0)	41 (35.7)	0.958^§^
Cough		7 (7.0)	21 (18.3)	0.014^§^
Newly diagnosed DM (n,%)		7 (7.0%)	11 (9.6%)	0.498^§^
Aetiology of DKA (n,%)	Poor adherence	48 (48.0)	36 (31.3)	
	Infection	24 (24.0)	35 (30.4)	
	Both poor adherence and infection	26 (26.0)	36 (31.3)	
	Others/Non-identified^*^	2 (2.0)	8 (7.0)	

The overall prevalence of hypoglycemia in our cohort is 23.7%. T1DM patients experienced a significantly higher rate of hypoglycemia during the initial management of DKA compared to T2DM patients. Conversely, hypokalemia rates were high in both groups, but there were no statistically significant differences (Table 3).

**Table 3:** Outcomes of DKA management by type of diabetes. IQR: interquartile range; ICU: intensive care unit; DKA: diabetic ketoacidosis *All-cause mortality, includes: septicemic shock with multiorgan failure, cardiogenic shock, acute coronary syndrome ^†^Pearson Chi-Square; ^‡^Mann-Whitney test; ^§^Fisher’s Exact test

Outcomes of DKA management	Type 1 Diabetes (N=100)	Type 2 Diabetes (N=115)	p value
Primary Outcomes			
Hypoglycemia events until DKA resolution or 48 hours (n,%)	33 (33.0)	18 (15.7)	0.003^†^
Hypokalemia events until DKA resolution or 48 hours (n,%)	55 (55.0)	62 (53.9)	0.873^†^
Secondary Outcomes			
Median (IQR) time to DKA resolution (hours)	18 (10.0)	17 (13.0)	0.337^‡^
Median (IQR) ICU length of stay (days)	2 (2.0)	2 (2.0)	0.273^‡^
Median (IQR) length of stay (days)	4 (3.0)	6 (5.0)	<0.001^‡^
ICU admission (n,%)	34 (34.0)	25 (21.7)	0.044^†^
Intubation (n,%)	11 (11.0)	17 (14.8)	0.411^†^
Nosocomial infections (n,%)	0 (0.0)	8 (7.0)	0.008^§^
Mortality during hospitalisation^*^ (n,%)	0 (0.0)	4 (3.5)	0.125^§^
All-cause-readmission within 30 days (n,%)	4 (4.0)	10 (8.7)	0.164^†^

T1DM patients had significantly higher Intensive Care Unit (ICU) admission rates, shorter overall hospital stays, and fewer nosocomial infections compared to T2DM patients. Mortality and readmission rate appeared worse in the T2DM group, though these did not reach statistical significance (Table 3). Among the 115 T2DM admissions, two (1.7%) had cardiac-related complications, two (1.7%) had venous thromboembolism, two (1.7%) required haemodialysis, and one (0.9%) had catheter-related bloodstream infection. Such complications were not observed in the T1DM group. All T1DM readmissions were due to recurrent DKA, compared to three (30.0% of readmissions) in the T2DM group. 

From the multivariate analysis, independent predictors of hypoglycemia are T1DM diagnosis and low body weight (Table 4). Hypokalemia independent predictors included lower initial serum potassium, lower pH, shorter diabetes duration, and absence of nephropathy (Table 5). 

**Table 4:** Hypoglycemia and its associated factors. *Forward likelihood ratio (LR) multiple logistic regression was applied. Forward LR method was performed for variables with p<0.250 in the multiple logistic regression analysis. Multicollinearity and interaction were checked and not found. Hosmer-Lemeshow test: p=0.397. Classification table: 76.3. Pseudo-R2 =0.100. T1DM: type 1 diabetes mellitus; T2DM: type 2 diabetes mellitus; DM: diabetes mellitus; CBG: capillary blood glucose; HCO3: bicarbonate; IV: intravenous; DKA: diabetic ketoacidosis; cOR: crude odds ratio; aOR: adjusted odds ratio; CI: confidence interval; HbA1c: hemoglobin A1c; OGLD: oral glucose lowering drug

Variables		Simple logistic regression				Multiple logistic regression^*^			
		cOR	95% CI		p value	aOR	95% CI		p value
			Lower	Upper			Lower	Upper	
Age (years)		0.993	0.977	1.009	0.400	-	-	-	-
Gender	Male	1	-	-	-	-	-	-	-
	Female	1.361	0.720	2.57	0.343	-	-	-	-
Diabetes type	T2DM	1	-	-	-	1	-	-	-
	T1DM	2.654	1.381	5.101	0.003	2.241	1.145	4.385	0.018
Body weight (kg)		0.964	0.940	0.990	0.006	0.970	0.945	0.996	0.023
DM duration	≤10 years	1	-	-	-	1	-	-	-
	>10 years	1.462	0.776	2.757	0.240	1.931	1.085	4.581	0.074
Nephropathy		0.423	0.198	0.905	0.027	0.523	0.227	1.208	0.129
Insulin and OGLDs		0.702	0.251	1.958	0.499	-	-	-	-
CBG (mmol/L)		0.974	0.946	1.003	0.081	0.970	0.938	1.004	0.086
pH		0.872	0.698	1.090	0.228	0.218	0.017	2.847	0.245
Serum ketone		1.010	0.991	1.028	0.311	-	-	-	-
Serum potassium (mmol/L)		1.104	0.800	1.524	0.546	-	-	-	-
Initial IV insulin dose (units/hour)		0.719	0.528	0.977	0.035	1.120	0.653	1.920	0.682
HbA1c (%)		0.953	0.855	1.063	0.386	-	-	-	-

**Table 5:** Hypokalemia and its associated factors. *Forward likelihood ratio (LR) multiple logistic regression was applied. Forward LR method was performed for variables with p<0.250 in the multiple logistic regression analysis. Multicollinearity and interaction were checked and not found. Hosmer-Lemeshow test: p=0.342. Classification table: 70.9. Pseudo-R2 =0.315. T1DM: type 1 diabetes mellitus; T2DM: type 2 diabetes mellitus; DM: diabetes mellitus; IV: intravenous; DKA: diabetic ketoacidosis; cOR: crude odds ratio; aOR: adjusted odds ratio; CI: confidence interval; HbA1c: hemoglobin A1c; OGLD: oral glucose lowering drug

Variables			Simple logistic regression				Multiple logistic regression^*^			
			cOR	95% CI		p value	aOR	95% CI		p value
				Lower	Upper			Lower	Upper	
Age (years)			0.974	0.960	0.988	<0.001	0.994	0.975	1.013	0.527
Gender		Male	1	-	-		1	-	-	-
		Female	1.569	0.914	2.694	0.103	1.509	0.740	3.075	0.258
Diabetes type		T2DM	1	-	-	-	-	-	-	-
		T1DM	1.045	0.610	1.790	0.873	-	-	-	-
Body weight (kg)			0.988	0.971	1.005	0.159	0.999	0.969	1.030	0.960
DM duration	≤10 years		1	-	-	-	1	-	-	-
	>10 years		0.259	0.145	0.460	<0.001	0.346	0.181	0.662	0.001
Nephropathy			0.419	0.234	0.752	0.004	0.462	0.237	0.899	0.023
Insulin and OGLDs			0.668	0.291	1.536	0.343	-	-	-	-
Diuretic use			1.263	0.207	7.716	0.800	-	-	-	-
pH			0.055	0.007	0.404	0.004	0.029	0.002	0.361	0.006
Serum ketone			1.000	0.988	1.011	0.965	-	-	-	-
Serum potassium (mmol/L)			0.447	0.321	0.622	<0.001	0.421	0.290	0.611	<0.001
Initial IV insulin dose (units/h)			0.795	0.615	1.028	0.080	0.922	0.596	1.424	0.713
HbA1c (%)			1.167	1.058	1.287	0.002	1.003	0.885	1.137	0.957

## Discussion

Our study revealed a marked divergence in hypoglycemic risk according to diabetes type. We observed a higher prevalence of hypoglycemia with T1DM compared to T2DM. Our findings align with Balmier et al., who reported significantly higher hypoglycemia rates in T1DM compared to T2DM during DKA treatment, proposing that management should be stratified by diabetes type [3]. This is consistent with the pathophysiology of T1DM, where severe insulin deficiency, defective counter-regulatory hormone responses and ongoing reliance on exogenous non-physiological insulin therapy create a narrow therapeutic safety margin [11]. The “one-size-fits-all” approach of fixed-rate insulin infusion (0.1units/kg/hour) appears to expose T1DM patients disproportionately to hypoglycemia, suggesting they may require lower infusion rates due to greater insulin sensitivity compared to the insulin-resistant T2DM cohort [1,3,8,12]. Current guidelines do not differentiate DKA management by diabetes type, largely due to the lack of concrete evidence from randomised controlled trials. A randomised controlled trial to study different insulin infusion rates is essential to refine DKA treatment protocols and reduce iatrogenic complications.

We found that T1DM diagnosis and low body weight are risk factors for developing hypoglycemia during DKA treatment. This aligns with previous studies where T1DM diagnosis and low BMI were identified as risk factors due to failure of hypoglycemia counter-regulation [13,14]. Ross et al. similarly identified low BMI as a significant risk factor for hypoglycemia during hyperglycemic crises treatment, with underweight (BMI<18.5kg/m2) patients having 4.5 increased odds [14]. Low weight may reflect a state of malnutrition, catabolism, and glycogen reserves depletion [14,15]. As T1DM is frequently associated with low body weight, these factors likely create a cumulative risk profile: the insulin sensitivity and defective counter-regulation of T1DM are further aggravated by the limited glycogen reserves and malnutrition associated with lower body weight.

We found no significant difference in hypokalemia prevalence, yet the absolute incidence was alarmingly high in our cohort. This is in contrast to several studies reporting higher hypokalemia rates in T2DM during DKA treatment [3,10], suggesting that other factors might contribute to precipitating hypokalemia. There is a high total body potassium deficit caused by gastrointestinal losses and osmotic diuresis in patients presenting with DKA, and insulin administration can shift potassium into cells rapidly [16,17]. This suggests that insulin-induced intracellular potassium shifts are universal in DKA treatment, regardless of the underlying diabetes pathology. This observation is supported by a recent multicentre study in China by Zhang et al., which found that 67.9% of DKA patients developed hypokalemia during treatment, despite protocol-guided replacement [16]. The high rates observed here and in other studies underscore that current protocols may be insufficient in matching the rapid potassium shifts driven by insulin and acidosis correction [1,3,16]. 

Our multivariate analysis highlighted lower initial serum potassium, lower initial pH, shorter diabetes duration, and the absence of nephropathy as independent predictors of hypokalemia. Lower serum potassium on admission may imply larger potassium deficits as reported in previous studies [12,16]. Low pH during DKA admissions could indicate more severe ketoacidosis and osmotic diuresis, leading to intracellular potassium depletion [16]. Severe acidosis drives a substantial efflux of potassium out of the cell and often masks true potassium deficit, which leads to delayed replacement [16,17]. The higher incidence of hypokalemia in patients without nephropathy and shorter diabetes duration is likely attributable to an intact renin-angiotensin-aldosterone system and preserved renal excretory function, which leads to effective potassium excretion during osmotic diuresis [16,18]. Conversely, we postulate that those with long-standing diabetes are more prone to potassium retention due to hyporeninemic hypoaldosteronism, a syndrome frequently associated with diabetic kidney disease that impairs aldosterone-mediated potassium secretion in the distal nephron [12,18].

In our cohort, T2DM patients presented with greater clinical complexity and had significantly longer hospital stays. This finding aligned with previous studies, likely contributed by older age, higher BMI and significantly more comorbidities such as nephropathy, cardiovascular, and cerebrovascular disease [3,6,7,10]. Eledrisi et al. reported a seven-fold increase in mortality and longer hospitalization for T2DM patients with DKA compared to T1DM, attributing this to older age and a higher burden of comorbidities such as cardiovascular and renal diseases [10]. These patients often require prolonged care to manage not just the resolution of ketoacidosis, but also treat the underlying precipitating factors (primarily infections) and their chronic comorbidities. This is further evidenced by the significantly higher rate of nosocomial infections observed in the T2DM group, likely contributing to the extended recovery times and highlighting the need for vigilant infection control in this group.

In our study, T1DM patients were significantly more likely to be admitted to the ICU; however, we observed a higher mortality rate in the T2DM group, which was not statistically significant. This seemingly contradictory finding may reflect systemic differences in healthcare and triaging practices where intensive care resources are prioritised for younger patients (predominantly T1DM) who are perceived to have a better reversible prognosis. Previous reports have suggested that T2DM patients with DKA are a vulnerable group, necessitating vigilant monitoring for complications beyond ketoacidosis, such as higher risk for inpatient mortality and long-term complications including stroke, dementia, major cardiovascular events, and chronic kidney disease [6,10]. Thus, the T2DM mortalities observed in our cohort are confounded by older age and the higher burden of concurrent illnesses. Moreover, because our sample size is not powered to detect mortality differences between T1DM and T2DM, the certainty of this observation is low and should be interpreted carefully. A larger case-control study adjusting for confounders is required to confirm the T2DM association with mortality.

As current DKA treatment recommendations are not tailored for risk factors, development of predictive indicators for hypoglycemia and hypokalemia will provide guidance for clinicians to refine the management of this diabetes emergency. Early identification of patients at risk of metabolic complications during DKA treatment enables personalized treatment planning and improve patient safety, especially in resource-limited facilities. In the future, technological advancement could further refine DKA treatments to reduce metabolic complications. Ongoing trials are evaluating the role of continuous glucose monitor (CGM) in paediatric T1DM with DKA, as well as development of a predictive model for hypokalemia during DKA management [16,19]. 

Limitations

This study relies on a review of medical records, which is limited by information bias, including diabetes misclassification, missing, incomplete, or inaccurate data. Being a single-centre study, the findings may reflect local population characteristics and resource constraints. Consequently, our results may have limited generalisability within Malaysia’s heterogeneous population. Additionally, the use of event-based rather than patient-based analysis could introduce unaccounted clustering effects, potentially resulting in overestimated precision. As a retrospective study, our findings are exploratory and do not imply causality, but are a valuable reference for future studies. Future case-control studies would be helpful to confirm the risk and generalise the observed differences in DKA metabolic complications and outcomes based on diabetes type.

## Conclusions

We demonstrated that T1DM patients managed according to existing protocol are at significantly higher risk of hypoglycemia, necessitating a more refined DKA management guideline. Although the prevalence of hypokalemia was not significantly different between the two groups, the high prevalence overall underscores the importance of close monitoring and reviewing current potassium replacement protocols. We also observed that T2DM patients represent a distinct, high-risk cohort characterised by longer hospital stays and increased susceptibility to nosocomial infections. Future guidelines should move toward incorporating diabetes-type-specific algorithms to optimise safety and reduce iatrogenic complications during the management of this life-threatening emergency, and comprehensive management for complex patient groups. Randomised controlled clinical trial could further confirm these preliminary findings, providing more evidence to enable guideline updates to address the unique needs of patients with T1DM and T2DM during DKA management.
